# Case report: Conversion therapy to permit resection of initially unresectable hepatocellular carcinoma

**DOI:** 10.3389/fonc.2022.946693

**Published:** 2022-10-06

**Authors:** Kang Chen, Cheng-Piao Luo, De-Xiang Ge, Ke-Lin Wang, Qin Luo, Yan-Zhi Li, Xue-Mei You, Bang-De Xiang, Le-Qun Li, Liang Ma, Jian-Hong Zhong

**Affiliations:** ^1^ Hepatobiliary Surgery Department, Guangxi Medical University Cancer Hospital, Nanning, China; ^2^ Pathology Department, Guangxi Medical University Cancer Hospital, Nanning, China; ^3^ Key Laboratory of Early Prevention and Treatment for Regional High Frequency Tumor (Guangxi Medical University), Ministry of Education, Nanning, China; ^4^ Guangxi Key Laboratory of Early Prevention and Treatment for Regional High Frequency Tumor, Nanning, China

**Keywords:** hepatocellular carcinoma, transarterial chemoembolization, immune checkpoint inhibitor, conversion therapy, tyrosine kinase inhibitor

## Abstract

Most patients with hepatocellular carcinoma (HCC) are diagnosed when the disease is already at an advanced stage, so they are not eligible for resection and their prognosis is poor. The combination of transarterial chemoembolization (TACE) with immune checkpoint inhibitors or tyrosine kinase inhibitors can improve unresectable HCC to the point that patients can be treated with surgery. Here we describe two cases of such “conversion therapy”. One patient was a 52-year-old man in Child-Pugh class A with treatment-naive HCC whose 11.3-cm tumor had invaded the middle hepatic vein and right branch of the portal vein. He was treated with TACE plus camrelizumab, and radical resection was performed 3 months later. No evidence of recurrence was observed during 5-month follow-up. The other patient was a 42-year-old man in Child-Pugh class A with HCC involving a 11.4-cm tumor and severe liver cirrhosis. The patient was treated with TACE and lenvatinib, but the embolic effect after one month was unsatisfactory, so the regional treatment was changed to hepatic artery infusion chemotherapy and transcatheter arterial embolization. Radical resection was performed 2 months later, and no recurrence was evident at 1-month follow-up. These cases demonstrate two conversion therapies that may allow patients with initially unresectable HCC to benefit from resection.

## Introduction

Globally, hepatocellular carcinoma (HCC) is the sixth most common malignancy and the third leading cause of cancer-related death (1). Surgery and liver transplantation are still the best radical treatments for HCC patients, which can provide good long-term survival. Unfortunately, about 70% of HCC patients are diagnosed at an advanced stage of the disease and are therefore ineligible for surgery (2). Recently, so-called “conversion therapies” have been described that can improve unresectable HCC enough that the patient can undergo resection, leading to much better prognosis (3, 4).

Several types of conversion therapy have been described, most often involving transarterial chemoembolization (TACE) (3). Immune checkpoint inhibitor (ICIs) and tyrosine kinase inhibitors (TKIs) have also proven promising for treating advanced HCC, alone and together (5, 6). Conversion therapies remain in the exploratory stage and there are no consensus standards.

Here we describe two patients with unresectable HCC in whom different types of conversion therapy proved effective at downgrading the cancer enough that the patients could be treated with resection, leading to recurrence-free survival.

## Case reports

### Case 1

**Figure 1:**
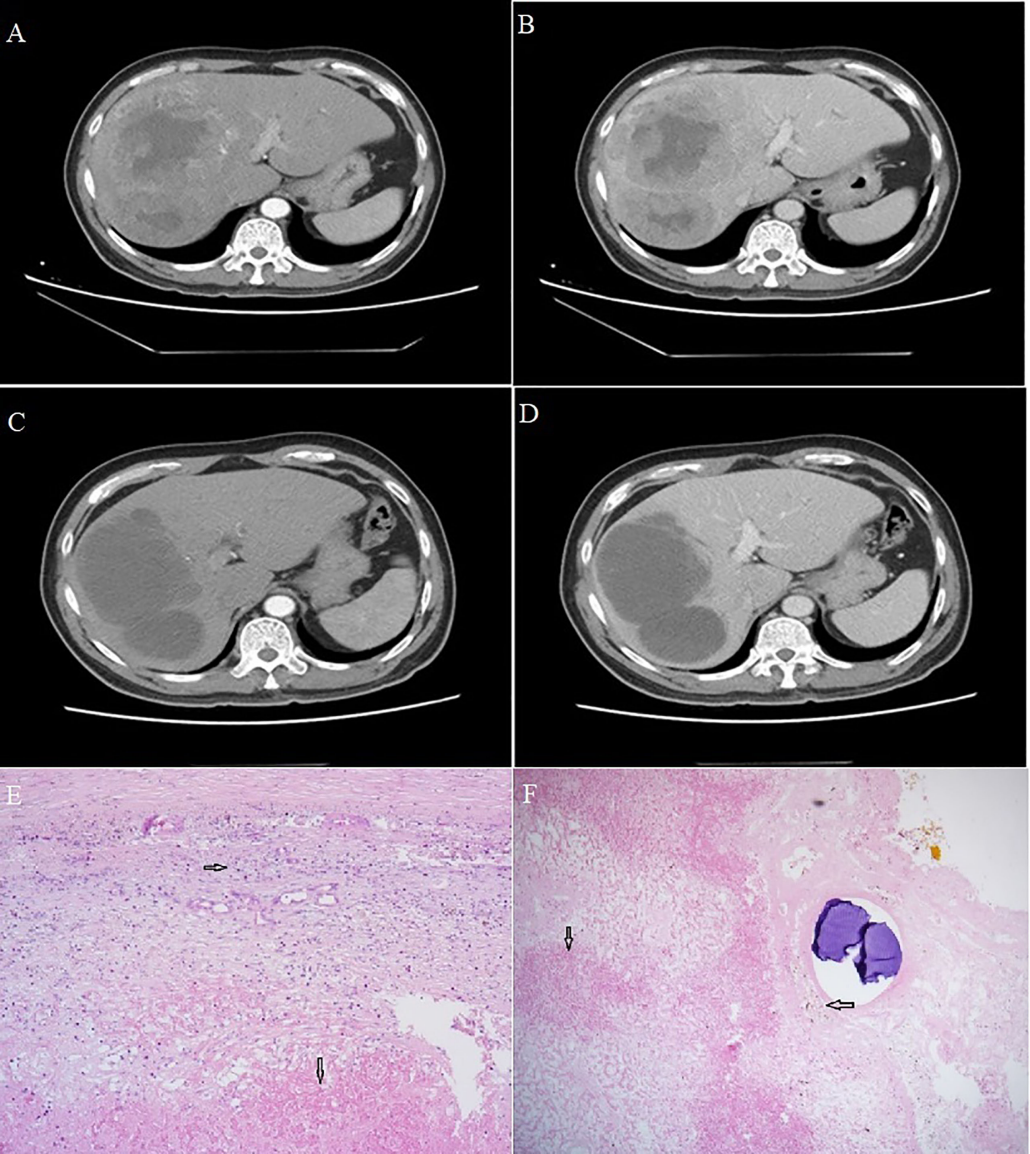
Computed tomography of Case 1. **(A, B)** Scans at admission showed a large, space-occupying lesion in the right liver, obvious inhomogeneous enhancement in the arterial phase, relatively low density in the portal phase, and a large area of non-enhancement in the tumor. **(C, D)** Scans after conversion therapy and before resection showed extensive necrosis in the primary lesion, with no residual activity in the arteriovenous phase. Sections of hepatocellular carcinoma tumor from Case 1 after conversion therapy and resection. **(E, F)** Visible are necrotic lesions (downward arrows), hyperplasia of surrounding fibrous tissue and lymphocyte infiltration (rightward arrow) and hemosiderin deposition (leftward arrow). Magnification, 40x.

A 52-year-old male patient was admitted to our hospital on May 22, 2021 due to pain in the right upper abdomen. He had been diagnosed with chronic infection with hepatitis B virus more than 20 years before, and he had a 10-year history of hypertension. Laboratory analysis revealed that the alpha-fetoprotein (AFP) level was 3.72 ng/ml, and the albumin level was 30.4 g/L (**Supplemental Table 1**). Dynamic enhanced computed tomography revealed multiple low-density shadows in the right lobe of the liver that were fused with one another (11.3 x 12.0 x 11.9 cm). Tumor invasion of the middle hepatic vein and right branch of the portal vein were observed, together with retroperitoneal lymph node metastasis (**Figures 1A, B**). The patient was assigned an Eastern Cooperative Oncology Group Performance Status (ECOG-PS) of 0, BCLC-C stage, Child-Pugh class of A and modified albumin-bilirubin (mALBI) stage of 2b.

The patient was initially given transarterial chemoembolization (TACE) involving 6 g eluting beads of pirarubicin (50 mg) as well as the PD-1 inhibitor camrelizumab (200 mg) once every 3 weeks for a total of nine weeks. The patient did not experience obvious adverse reactions, except mild fever during the night following TACE. On August 21, 2021, dynamic computed tomography showed no significant change in the size of multiple lesions in the liver, but extensive necrosis of lesions was observed, without obvious enhancement (**Figures 1C, D**). AFP at this time was 2.06 ng/ml, still within the normal range. Given the apparent success of the conversion therapy, the patient was treated by open right hemihepatectomy and cholecystectomy on August 27, 2021. Postoperative pathology showed coagulative necrosis of all hepatic tumors, hyperplasia of surrounding fibrous tissue, lymphocyte infiltration, and no residual cancer cells (**Figures 1E, F**).

After surgery, the patient continued to receive camrelizumab once every 3 weeks for a total of 15 weeks. During follow-up until July 10, 2022, no tumor recurrence was detected based on computed tomography or AFP.

### Case 2

**Figure 2:**
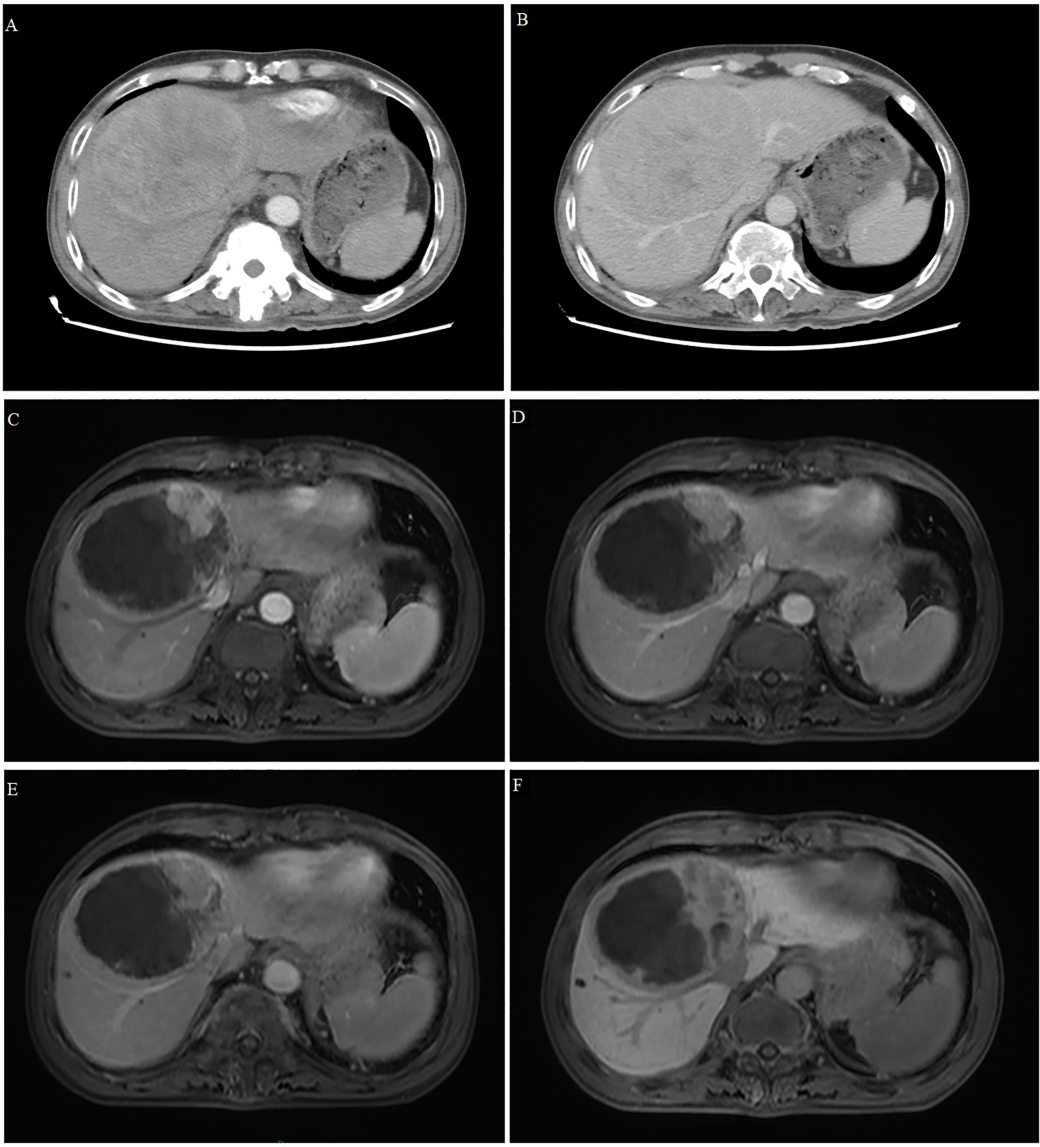
Computed tomography and magnetic resonance imaging of Case 2. **(A, B)** Tomography scans at admission showed a large, space-occupying lesion in the right liver, obvious inhomogeneous enhancement in the arterial phase **(A)** and relatively low density in the portal phase **(B)**. **(C–F)** T1-weighted magnetic resonance imaging after conversion therapy and before resection showed extensive tumor necrosis, but there was still a small active area around the tumor showing high signal intensity in the early arterial phase **(C)** and low signal intensity in the late arterial phase **(D)**, portal vein phase **(E)** and hepatobiliary phase **(F)**.

A 42-year-old male patient was admitted to our hospital on August 27, 2021 after dynamic computed tomography revealed a lump in the right lobe of the liver and AFP was found to be elevated. For more than 30 years, the patient had had cirrhosis associated with hepatitis B virus infection. AFP on admission was 992.8 ng/ml, and albumin was 31.5 g/L (**Supplementary Table**). Dynamic enhanced computed tomography revealed a lesion (11.4 x 8.9 x 10.0 cm) on the inferior segment of the right anterior lobe without macrovascular invasion or extrahepatic metastases, but with liver cirrhosis and splenomegaly with collateral circulation (**Figures 2A, B**). The patient was assigned an ECOG-PS score of 0, BCLC-A stage, Child-Pugh class of A and mALBI stage of 2b.

Given the patient’s large tumor, cirrhosis and < 45% residual liver volume, he was not considered eligible for surgery. After the absence of contraindications was confirmed, the patient was given superselective TACE involving raltitrexed (4 mg) and oxaliplatin (100 mg) as an emulsion in 40% iodized poppy oil (10 ml), as well as the tyrosine kinase inhibitor lenvatinib (8 mg, once daily). The patient experienced no obvious adverse reactions during treatment, except mild fever. One month later, dynamic computed tomography revealed that the lesion had shrunk slightly (9.9 x 7.4 cm), about half the lipiodol in the lesion had washed away, and the area without lipiodol deposition still showed partial enhancement. AFP at this time was 140.33 ng/ml. This suggested inadequate embolization by TACE, so the patient was switched to hepatic arterial infusion chemotherapy (HAIC) plus transcatheter arterial embolization. After 3 days of HAIC involving calcium leucovorin (600 mg), fluorouracil (4.0 mg) and oxaliplatin (200 mg), the patient underwent transcatheter arterial embolization. During treatment, the patient experienced no serious adverse events, except for mild fever. The patient was discharged and given lenvatinib therapy for 2 months.

**Figure 3:**
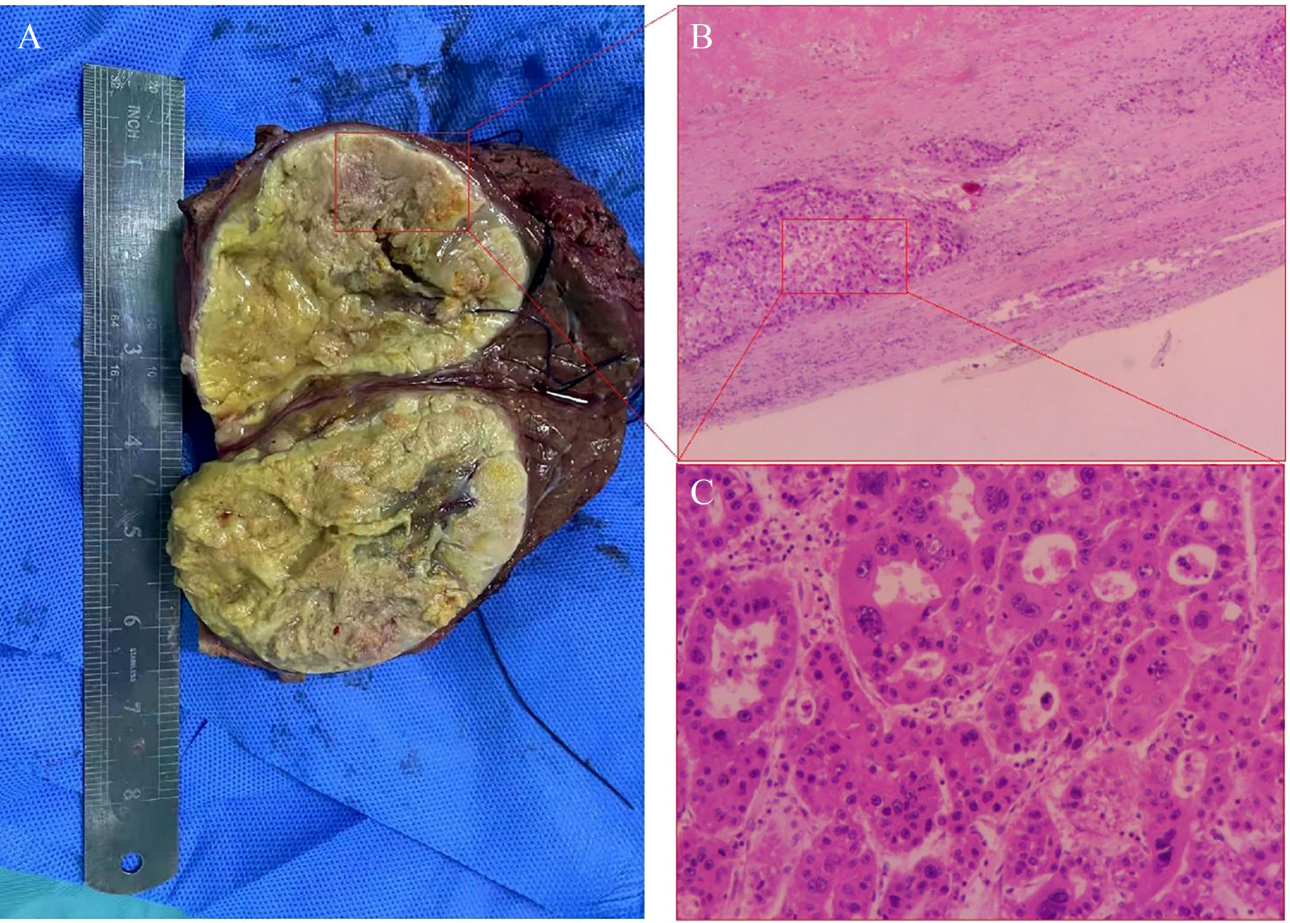
Histopathology of tumor tissue in Case 2 after conversion therapy and resection. **(A)** Viable cancer cells were observed (inside the red box), as well as necrotic cells (outside the red box). **(B)** The primary lesion showed massive necrosis with lymphocyte infiltration, and scattered tumor cell nests surviving around the lesion (red box). Magnification, 40x. **(C)** Higher-magnification image of the red box in panel **(B)** shows some degenerated tumor cells and some giant tumor cells. Magnification, 100x.

On December 1, 2021, dynamic enhanced magnetic resonance imaging showed that the tumor had shrunk substantially (9.5 x 7.9 x 8.7 cm), the original lesion showed extensive necrosis, and some active lesions were situated around the original one (**Figures 2C-F**). AFP was 70.06 ng/ml at this time. The apparent success of the conversion therapy and the patient’s strong desire for surgery led to open right liver tumor radical resection and cholecystectomy on December 10, 2021. Histopathology showed coagulative necrosis, a few surviving cancer cells around the tumor, and some degenerated cancer cells. The lesion area also showed substantial fibrous hyperplasia with lymphocyte infiltration (**Figure 3**). No tumor recurrence was detected during follow-up through July 5, 2022, based on computed tomography and AFP (**Supplemental Figure S1**).

## Discussion

**Figure 4:**
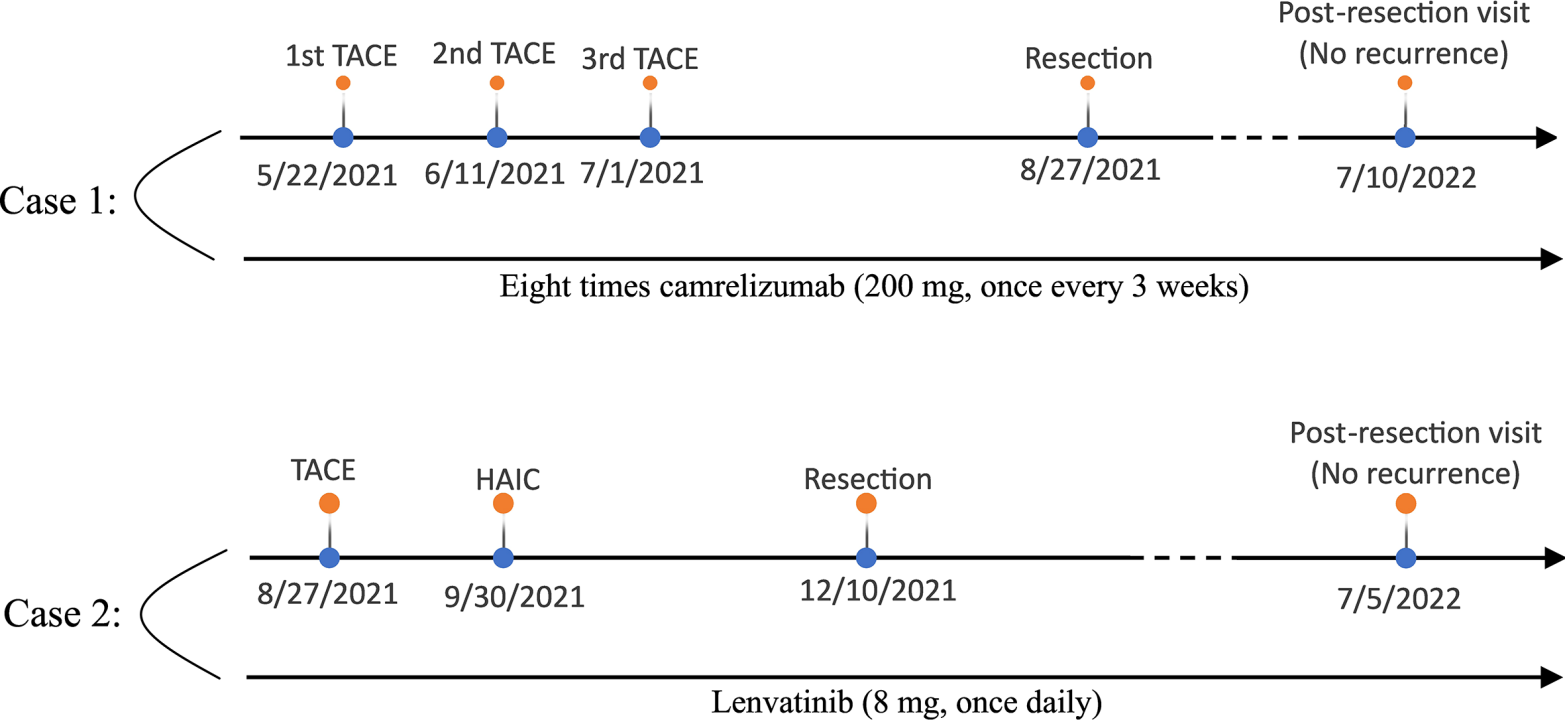
The treatment timeline of the two patients (top: case 1; below: case 2).

The two cases in this report demonstrate that local regional therapy plus TKIs or ICIs can improve initially unresectable HCC enough that patients can undergo resection and have better prognosis (**Figure 4**).

First-line treatment of unresectable HCC is usually TACE and atezolizumab-bevacizumab or durvalumab-tremelimumab. If the above treatment fails, other options include regorafenib, cabozantinib, and remolumab (7). On their own, TKIs or ICIs are associated with objective response rates only around 20% (5), while combining immune drugs with targeted drugs can improve objective response (4, 6, 8). For example, lenvatinib combined with pembrolizumab in one trial led to median progression-free survival (mPFS) of 9.3 months and median overall survival (mOS) of 22 months in patients with unresectable HCC (9). In addition, ORIENT-32 study found that sintilimab plus bevacizumab showed a significant mPFS and mOS benefit versus sorafenib for patients with unresectable, HBV-associated HCC (10). Similarly, this study chose the combined treatment of local regional therapy plus immunotherapy, all the patients achieved tumor downstaging and the opportunity of surgical resection, and theoretically they could get longer OS. This combination regimen may provide a novel treatment option for unresectable HCC patients.

Pembrolizumab showed a high objective response rate in clinical trials and therefore was the first PD-1 inhibitor to be approved for clinical use (11). Its high cost in mainland China led our Case 1 to opt for the locally produced PD-1 inhibitor camrelizumab, which has shown similar efficacy to pembrolizumab (12). In that patient’s conversion therapy, TACE presumably killed tumor cells by embolizing the tumor and causing cytotoxicity because of the chemotherapeutics, while camrelizumab restored endogenous anti-tumor T cell responses and induced tumor cell apoptosis (13). In this way, TACE and camrelizumab exerted synergistic anti-tumor effects.

HAIC can continuously infuse high concentrations of cytotoxic drugs into tumor-associated arteries, leading to strong anti-tumor effects without significantly damaging the liver. Trials have reported that HAIC involving fluorouracil, calcium folinate and oxaliplatin led to median progression-free survival of 7.8 months and median overall survival of 13.9 months, much better than the corresponding survival times of 4.3 and 8.2 months for sorafinib (14, 15). In fact, the Chinese Society of Clinical Oncology recommends this HAIC regime for advanced HCC. We selected a conversion regime for Case 2 involving TACE, lenvatinib and HAIC. We attribute the efficacy of this approach to several effects: (1) TACE and HAIC induce tumor ischemia and necrosis, leading to direct anti-tumor effects; (2) the small-molecule kinase inhibitor lenvatinib prevents this ischemia and hypoxia from upregulating vascular endothelial growth factor, fibroblast growth factor and platelet-derived growth factor, in turn inhibiting angiogenesis and thereby leading to direct as well as indirect anti-tumor effects; and (3) lenvatinib may normalize tumor vessels, facilitating the distribution and delivery of anticancer drugs such as pirarubicin. However, at present, both TACE and HAIC are local regional therapy schemes for HCC, and there are no official guidelines to determine which treatment is the best local treatment. Therefore, we are carrying out relevant clinical research to explore which treatment is the best treatment.

Some studies have shown that for cirrhotic patients with HCC, laparoscopic liver resection (LLR) is superior to open liver resection in perioperative safety and postoperative recovery time, and there is no significant difference in OS (16, 17). In addition, LLR can reduce postoperative abdominal adhesion and provide opportunities for reoperation or salvage liver transplantation after tumor recurrence, further prolonging OS (18). However, the tumor of the two patients in this study was large (>10cm) and located in an unfavorable resection position (right anterior segment), the LLR was difficult and had no advantage in reducing the incidence of postoperative complications, so the two patients finally chose open liver resection.

According to the XXL trial, if the HCC patients beyond the Milan criteria achieve partial or complete response after tumor downstaging, the prognosis of liver transplantation is better than that of continuous systemic therapy (19). In addition, for cirrhotic patients with HCC, liver transplantation can completely cure liver cirrhosis, so the prognosis of liver transplantation is better than that of liver resection. Considering the advantages of liver transplantation over liver resection or systemic treatment, it may be better for patients in this study to choose liver transplantation after liver tumor recurrence, especially for cirrhotic patients with HCC.

How best to evaluate the efficacy of conversion therapy is unclear. The imaging-based evaluation criteria RECIST and mRECIST are commonly used to examine treatment response in HCC. Unlike RECIST, mRECIST focuses on blood supply to the tumor rather than tumor size. We applied mRECIST criteria to both patients in our study, and we found the postoperative assessment to be partial or complete response, consistent with other laboratory and histopathological indicators that we examined. Our results suggest that mRECIST may be accurate and effective for evaluating the efficacy of conversion therapy, which should be explored in further clinical studies.

In Case 2, AFP decreased substantially during treatment, suggesting that the level may be useful for evaluating the efficacy of conversion therapy. If AFP does not change during conversion therapy, applying other targeted therapy or immunotherapy may be beneficial. Resected surgical specimens from both our patients showed lymphocyte infiltration. It would be interesting to examine whether this observation is associated with the good prognosis that we observed for both patients after conversion therapy.

## Conclusions

These two cases indicate that for patients with normal liver function, combining local regional therapy of TACE or/and HAIC with ICIs or TKIs may be a tolerable and effective way to render initially unresectable HCC amenable to surgery – even radical surgery – that may improve long-term survival. Our clinical experience should be explored in cohort studies.

## Data availability statement

The original contributions presented in the study are included in the article/**Supplementary material**, further inquiries can be directed to the corresponding author/s.

## Ethics statement

Written informed consent was obtained from the individual(s) for the publication of any potentially identifiable images or data included in this article.

## Author contributions

J-HZ performed the research. KC and J-HZ designed the study and wrote the paper. All authors approved the final version of the manuscript.

## Funding

This work was supported by the Specific Research Project of Guangxi for Research Bases and Talents (GuiKe AD22035057), the National Natural Science Foundation of China (82060510 and 82260569), the Guangxi Undergraduate Training Program for Innovation and Entrepreneurship (X202210598347), and the Key Laboratory of Early Prevention and Treatment for Regional High Frequency Tumor (Gaungxi Medical University), Ministry of Education (GKE-ZZ202217).

## Conflict of interest

The authors declare that the research was conducted in the absence of any commercial or financial relationships that could be construed as a potential conflict of interest.

## Publisher's note

All claims expressed in this article are solely those of the authors and do not necessarily represent those of their affiliated organizations, or those of the publisher, the editors and the reviewers. Any product that may be evaluated in this article, or claim that may be made by its manufacturer, is not guaranteed or endorsed by the publisher.

## Supplementary material

Supplementary Figure 1 Alpha-fetoprotein levels in Case 2 at different stages of treatment. HAIC, hepatic arterial infusion chemotherapy.
