# Supplementary material for: Case report: Conversion therapy to permit resection of initially unresectable hepatocellular carcinoma
Source: Front Oncol. 2022 Oct 6;12:946693. doi: 10.3389/fonc.2022.946693 (PMC9583878; doi:10.3389/fonc.2022.946693)
Supplement: Supplementary file 1 [file Table_1.docx]

**Supplementary Table.**  Baseline characteristics of the two patients.

| Characteristics | Case 1 | Case 2 |
| --- | --- | --- |
| Sex | Male | Male |
| Age, yr | 52 | 42 |
| ECOG performance status | 0 | 0 |
| Etiology of HCC | HBV | HBV |
| BCLC stage | C | C |
| China liver cancer stage | IIIa | Ib |
| Macrovascular invasion | Yes | No |
| Portal vein tumor thrombus | Yes | No |
| Extrahepatic disease | No | No |
| Child-Pugh class | A | A |
| Modified albumin-bilirubin stage | 2b | 2b |
| Alpha-fetoprotein, ng/ml | 3.72 | 992.8 |
| Alanine transaminase, U/L | 38 | 31 |
| Aspartate aminotransferase, U/L | 24 | 28 |
| Albumin, g/L | 30.4 | 31.5 |

BCLC, Barcelona Clinic liver cancer; ECOG, Eastern Cooperative Oncology Group; HCC, hepatocellular carcinoma.
