# Supplementary figures and images for: Case report: Conversion therapy to permit resection of initially unresectable hepatocellular carcinoma
Source: Front Oncol. 2022 Oct 6;12:946693. doi: 10.3389/fonc.2022.946693 (PMC9583878; doi:10.3389/fonc.2022.946693)

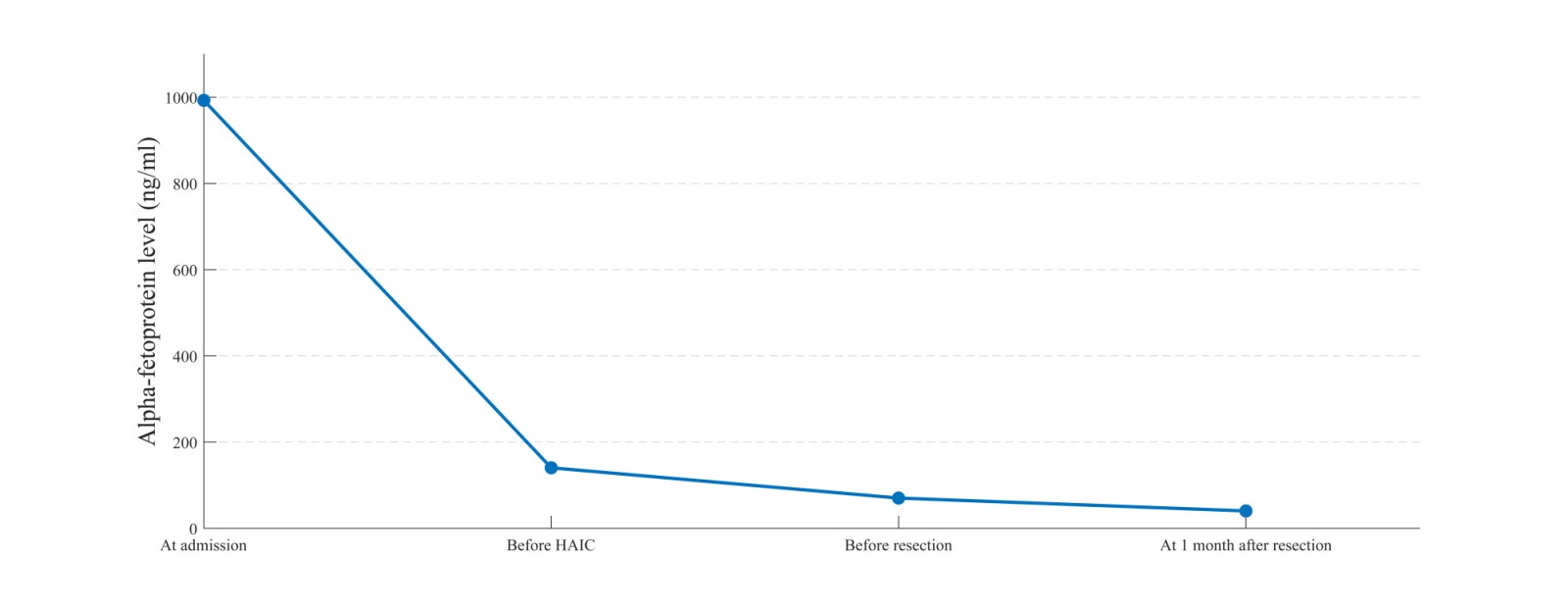

Supplement: Supplementary Figure 1 — Alpha-fetoprotein levels in Case 2 at different stages of treatment. HAIC, hepatic arterial infusion chemotherapy. [file Image_1.jpeg]
